# Awareness of Stroke and Preventive Measures Among Hypertensive Patients in a Tertiary Care Hospital: A Cross-Sectional Survey

**DOI:** 10.7759/cureus.83222

**Published:** 2025-04-29

**Authors:** Natwarlal Patidar, David Ratna Paul Talagatoti, Ravi Gaur, Imran Khan, Nitika Thakur

**Affiliations:** 1 College of Nursing, All India Institute of Medical Sciences, Jodhpur, Jodhpur, IND; 2 Nursing, Sharda School of Nursing Science and Research, Sharda University, Greater Noida, IND; 3 Physical Medicine and Rehabilitation, All India Institute of Medical Sciences, Jodhpur, Jodhpur, IND; 4 Pediatric Nursing, Sharda School of Nursing Science and Research, Sharda University, Greater Noida, IND

**Keywords:** a cross-sectional study, awareness, hypertension, preventive measures, stroke

## Abstract

Introduction: Stroke is a major cause of death, with hypertension being a significant modifiable risk factor. Recognizing stroke symptoms early and receiving prompt treatment can greatly reduce both morbidity and mortality. Evaluating stroke awareness among hypertensive patients is crucial for developing effective health promotion strategies. This study was conducted with the primary objective to assess the level of stroke awareness and preventive practices among individuals with hypertension.

Materials and methods: The study was conducted at All India Institute of Medical Sciences, Jodhpur, a tertiary care teaching hospital in western Rajasthan, India, using a cross-sectional study design. The ethical clearance was received from Institutional Ethical Clearance committees. This study was conducted over a three-month period, from May to July 2023. Among all patients who attended the outpatient department of the neurology and cardiology department, 324 patients were selected using the nonprobability purposive sampling technique for the study who were fulfilling inclusion criteria. A self-structured awareness questionnaire tool was used for data collection. The questionnaire consisted of 24 items, with each item carrying a score of 1 point. The questionnaire has five domains that include the meaning of stroke, risk factors and causes, warning signs and symptoms, diagnostic tests, and treatment and prevention. The total score of the questionnaire was classified into three levels: poor knowledge (0-8), fair knowledge (9-16), and good knowledge (17-24).

Results: The study assessed stroke awareness among 324 hypertensive patients. Awareness of the definition of stroke was observed in 55% of participants. Knowledge of risk factors and causes was relatively low, with only 31.25% of participants demonstrating adequate understanding. In contrast, awareness of stroke warning signs and symptoms was notably higher, with 73.7% of participants showing good awareness. Awareness of diagnostic tests was moderate, with 50.5% of respondents correctly identifying relevant information. The highest level of knowledge was seen in the area of treatment and prevention, where 75.7% of participants displayed a satisfactory understanding. Categorizing their knowledge levels as poor, fair, or good. A total of 135 patients (41.7%) demonstrated a fair level of awareness, with an average score of 13.84±5.92. Additionally, 102 patients (31.5%) exhibited good awareness, while 87 patients (26.9%) had poor awareness. Most participants (41.66%) demonstrated a fair level of awareness, with a mean score of 13.84±5.92. Approximately 31.48% of patients exhibited good awareness, while 26.85% had poor awareness.

Conclusion: Although hypertensive patients demonstrated a general awareness of stroke, their knowledge regarding specific risk factors and warning signs was limited, and engagement in stroke prevention behaviors was inadequate. These findings underscore the necessity for structured, evidence-based educational interventions to improve knowledge, foster appropriate attitudes, and address misconceptions related to stroke prevention among individuals with hypertension.

## Introduction

In 2021, India bore a substantial share of the global stroke burden, contributing to 10% of the worldwide total. Globally, strokes affected 11.9 million people - a 70% rise since 1990. Within India, there were 1.25 million new stroke cases reported in 2021, representing a 51% increase from 1990. This sharp rise in stroke incidence in India plays a major role in the growing global impact of the disease [1]. As of 2023, India has witnessed a 2.3-fold increase in the prevalence of ischemic heart disease and stroke over the past three decades, escalating from 25.7 million cases in 1990 to 64 million cases in 2023 [2].

Stroke, in particular, is a significant global health issue and remains the leading cause of death in low- and middle-income countries. Early recognition of stroke symptoms and prompt treatment can drastically reduce both the severity and mortality of stroke. More than 90% of the stroke burden is associated with modifiable risk factors, which include behavioral and environmental factors [3]. Common risk factors for stroke include conditions such as hypertension, diabetes mellitus, hyperlipidemia, atrial fibrillation, and lifestyle factors like smoking. Among these, hypertension stands out as the most consistently recognized modifiable risk factor for stroke. Addressing these risk factors through prevention and management strategies could significantly reduce the incidence and impact of stroke globally [4].

Stroke prevention is not only feasible but also highly effective, with studies showing that up to 80% of strokes can be prevented through timely and appropriate actions based on understanding the disease's risk factors [5]. The key to prevention lies in increasing awareness about these risk factors and the importance of early detection and management of stroke symptoms. When primary prevention strategies and medical interventions are implemented promptly, they can save lives [6].

Over the past four decades, stroke incidence has more than doubled in low- and middle-income countries, while it has decreased by 42% in high-income countries. Additionally, deaths due to stroke have been significantly higher in low- and middle-income countries over the last 15 years compared to their high-income counterparts [7]. Beyond the physical health consequences, stroke has a profound impact on individuals’ productivity and quality of life, which in turn affects the socio-economic development of nations [8].

Despite the global burden of stroke, many hypertensive patients remain insufficiently informed about its causes, risk factors, prevention, and treatment. Public awareness of stroke warning signs plays a critical role in ensuring timely recognition and intervention, which are key to improving outcomes and reducing complications [9]. Over the past decade in India, initiatives to promote stroke prevention among individuals with hypertension have concentrated on enhancing knowledge, attitudes, and practices, especially within high-risk populations. These efforts have involved tailored health education programs, the use of media and community outreach campaigns, and a strong emphasis on lifestyle modifications such as lowering salt consumption and encouraging regular physical activity [10].

This survey was conducted to assess baseline stroke knowledge among hypertensive patients, providing crucial insights for developing targeted and effective health promotion campaigns focused on stroke prevention. The primary objectives of this study are to assess stroke-related knowledge of risk factors, early signs, prevention, diagnosis, and treatment among hypertensive outpatients at a tertiary care facility in India.

## Materials and methods

The study was conducted at All India Institute of Medical Sciences, Jodhpur, a tertiary care teaching hospital in western Rajasthan, India, using a cross-sectional study design. The ethical clearance was received from the Institutional Ethical Committee, All India Institute of Medical Sciences (AIIMS), Jodhpur, with letter no. AIIMS/IEC/2023/9905 dated March 6, 2023. This study was conducted over a three-month period, from May to July 2023. Among all patients who attended the outpatient department of the neurology and cardiology department, 324 patients were selected using the nonprobability purposive sampling technique for the study who were fulfilling inclusion criteria. The sample size of 324 hypertensive patients was determined based on the formula for cross-sectional studies with the Z-score for a 95% confidence interval (1.96), assumed prevalence of adequate stroke awareness among hypertensive patients (taken as 30% based on previous literature), with the margin of error set at 5%. Considering a 10% non-response rate, the final calculated sample size was approximately 324.

The study included hypertensive patients who met the following criteria: they were over 20 years old, had been diagnosed with hypertension at least six months prior, were willing to participate, and were able to understand the study language, which is Hindi or English. Patients were excluded if they had a history of stroke, brain tumor, head injury, intellectual disability, mental illness, etc.

The data collection tool included two sections. Section I is the socio-demographic characteristic sheet. Part A of the socio-demographic data sheet comprised personal details of participants that included age, gender, marital status, education, and social background, etc., and Part B comprised a questionnaire about the comprehensive clinical history of participants that included duration of hypertension, duration of treatment, family history of hypertension, body mass index (BMI), previous information about stroke, and any other comorbidities. Section II comprised a self-structured awareness questionnaire used for data collection. The content validity index, calculated from the scores of seven experts, was ≥74 for the awareness questionnaire. Also, reliability has been checked for internal consistency by using Cronbach’s alpha, and the score was found to be 0.806. The questionnaire consisted of 24 items, with each item carrying a score of 1 point. The tool has been translated into the Hindi language for a better understanding of participants. The questionnaire has five domains that include the meaning of stroke, risk factors and causes, warning signs and symptoms, diagnostic tests, and treatment and prevention. The total score of the questionnaire was classified into three levels: poor knowledge (0-8 score), fair knowledge (9-16 score), and good knowledge (17-24 score). A pilot study, involving 10% of the sample size for the main study, was conducted to test the feasibility of the data collection process.

Data were analyzed using IBM SPSS Statistics for Windows, Version 20 (Released 2011; IBM Corp., Armonk, New York, United States). Descriptive statistics, including frequency, percentage, mean, and standard deviation, were employed to summarize the socio-demographic characteristics of the hypertensive patients. Inferential statistics, specifically the chi-square test, were used to assess associations between awareness levels and socio-demographic variables.

## Results

Demographic characteristics

The mean age of hypertensive patients was 55.65±14.51 years. Among the participants, 58.6% were male, while 41.3% were female. Table 1 shows the demographic characteristics of the study subjects (n=324).

**Table 1:** Demographic characteristics of study subjects (n=324)

Personal characteristics	Participants (%)
Age	
21-40 years	24 (7.40)
41-60 years	198 (61.11)
>60 years	102 (31.48)
Mean±SD	55.65±14.51
Gender	
Male	190 (58.64)
Female	134 (41.35)
Marital status	
Unmarried	13 (4.01)
Married	266 (82.10)
Widow/widower	45 (13.88)
Education	
No formal education	114 (35.18)
Primary	62 (19.13)
Secondary	103 (31.80)
Graduation and above	45 (13.88)
Occupation	
Home maker	121 (37.34)
Unemployed	27 (8.33)
Self-employed	84 (25.92)
Private employee	51 (15.74)
Government employee	38 (11.73)
Income per month in Indian Rupees	
<10,000	39 (12.03)
10,000-20,000	128 (39.50)
20,000-50,000	96 (29.62)
>50,000	61 (18.82)
Social background	
Rural	179 (55.24)
Urban	145 (44.75)
Clinical variables	Participants (%)
Duration of hypertension	
6 month-1 year	68 (20.98)
2-5 years	121 (37.34)
6-10 years	103 (31.79)
>10 years	32 (9.87)
Duration of treatment	
<1 year	48 (14.81)
2-5 years	132 (40.74)
6-10 years	118 (36.42)
>10 years	26 (8.02)
Co-morbidity of kidney diseases and diabetes mellitus	
Yes	134 (41.35)
Diabetes mellitus	81 (60.44)
Kidney disease	32 (23.88)
Both co-morbidity	21 (15.67)
No	190 (58.64)
Family history of hypertension, kidney diseases, and diabetes mellitus	
Yes	185 (57.10)
Diabetes mellitus	71 (38.37)
Kidney disease	16 (8.64)
Hypertension	98 (52.97)
No	139 (42.90)
Body mass index	
<18.5 (underweight)	17 (5.24)
18.1-24.9 (normal weight)	194 (59.87)
25-29.9 (overweight)	89 (27.46)
30-34.9 (class-I obesity)	24 (7.40)
Previous knowledge regarding stroke	
Yes	222 (68.51)
No	102 (31.48)

Level of awareness regarding stroke in hypertensive patients

**Figure 1:**
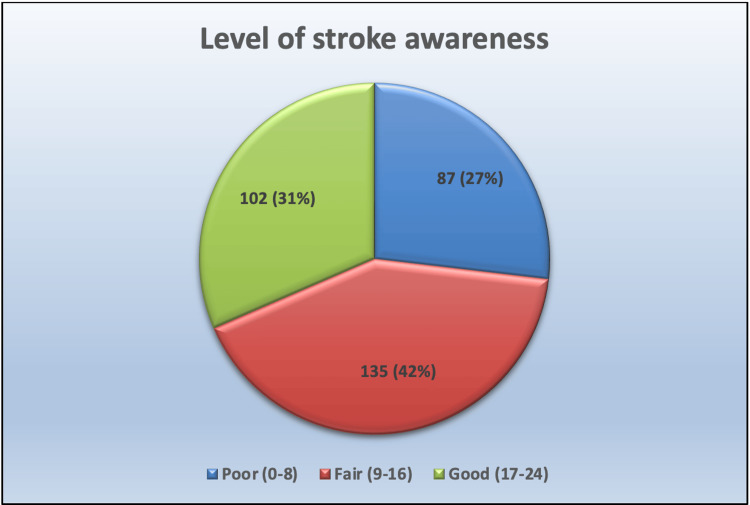
Pie chart indicating the level of stroke awareness among hypertensive patients

Table 2 summarizes the level of stroke awareness among 324 hypertensive patients, categorized as poor, fair, or good. Most participants (41.66%) demonstrated a fair level of awareness, with a mean score of 13.84±5.92. Approximately 31.48% of patients exhibited good awareness, while 26.85% had poor awareness (Figure 1). This distribution highlights the need for targeted interventions to improve stroke awareness, especially among those with limited knowledge.

**Table 2:** Level of awareness regarding stroke in hypertensive patients (n=324)

Level of stroke awareness	f-value	Percentage	Mean±SD
Poor (0-8)	87	26.85	
Fair (9-16)	135	41.66	13.84±5.92
Good (17-24)	102	31.48	

**Figure 2:**
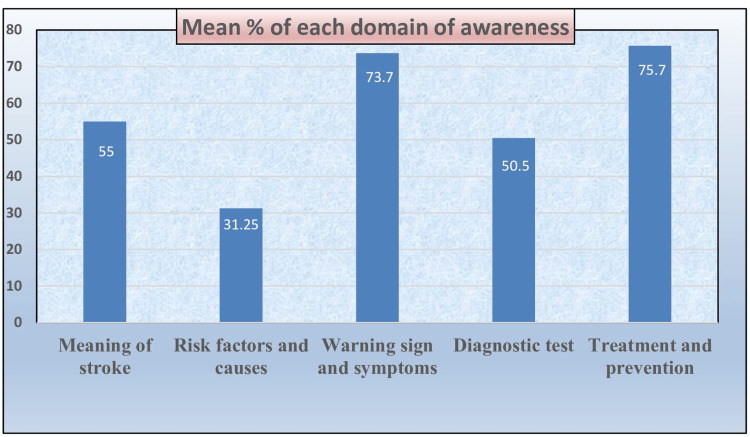
Bar graph indicating the mean percentage of awareness across different domains

Table 3 provides a summary of various domains of stroke awareness among participants, along with the corresponding number of items, mean scores, and percentage awareness. Awareness of stroke meaning scored 1.1±0.63 (55%), while knowledge of risk factors and causes was relatively low, scoring 3.12±3.37 (31.25%). Awareness of warning signs and symptoms was notably higher, with a mean score of 4.42±2.61 (73.7%). Diagnostic test awareness was moderate at 0.50±0.50 (50.5%), and treatment and prevention awareness was the highest, scoring 3.78±1.41 (75.7%) (Figure 2). This data indicates variability in knowledge across different domains, with prevention and symptoms being better understood than causes or diagnostic tests.

**Table 3:** Domain-wise mean, standard deviation, and mean percentage of awareness

S. No.	Domains	No. of items	Mean±SD	Mean (%)
1	Meaning of stroke	1-2 (2)	1.1±0.63	55
2	Risk factors and causes	3-12 (10)	3.12±3.37	31.25
3	Warning signs and symptoms	13-18 (6)	4.42±2.61	73.7
4	Diagnostic test	19 (1)	0.50±0.50	50.5
5	Treatment and prevention	20-24 (5)	3.78±1.41	75.7

Association of level of awareness regarding stroke in hypertensive patients with personal and clinical characteristics

The findings of the study (Table 4) show a significant association between stroke awareness among hypertensive patients and age, occupation, education, social background, duration of hypertension, BMI, and previous knowledge at the p < 0.05 level of significance (n=324).

**Table 4:** Association of level of awareness regarding stroke in hypertensive patients with personal and clinical characteristics * indicates p<0.05 (level of significance); ^NS ^indicates not significant

Personal characteristics	Level of awareness			X^2^	df	p-value
	Poor	Fair	Good			
Age						
21-40 years	10	6	8			
41-60 years	51	67	80	9.57	4	0.03*
>60 years	28	43	29			
Gender						
Male	49	72	69	0.45	2	0.82^NS^
Female	34	52	48			
Marital status						
Unmarried	4	6	3			
Married	67	104	95	1.85	4	0.78^NS^
Widow/widower	10	21	15			
Education						
No formal education	39	47	28			
Primary	20	24	18	42.73	8	0.00*
Secondary	27	41	35			
Graduation and above	9	17	19			
Occupation						
Home maker	38	49	34			
Unemployed	7	11	9	38.67	8	0.00*
Self-employed	23	36	25			
Private employee	10	19	22			
Government employee	8	16	14			
Income per month						
<10,000	15	14	10			
10,000-20,000	42	55	31	13.23	6	1.92^NS^
20,000-50,000	30	35	31			
>50,000	17	21	23			
Social background						
Rural	58	76	45	21.75	2	0.00*
Urban	32	46	67			
Clinical variables						
Duration of hypertension						
6 month-1 year	17	28	23			
2-5 years	46	43	32	5.76	6	0.04*
6-10 years	35	38	29			
>10 years	11	14	7			
Duration of treatment						
<1 year	17	15	10			
2-5 years	43	54	35	23.17	6	2.78^NS^
6-10 years	37	52	29			
>10 years	9	11	6			
Co-morbidity of kidney diseases and diabetes mellitus						
Yes	43	52	39	9.42	2	0.91^NS^
No	53	75	62			
Family history of hypertension, kidney diseases and diabetes						
Yes	48	73	64	21.43	2	1.67^NS^
No	47	57	35			
Body mass index						
<18.5 (underweight)	9	3	5			
18.1-24.9 (normal weight)	46	80	68	13.65	6	0.03*
25-29.9 (overweight)	23	35	31			
30-34.9 (class-I obesity)	11	7	6			
Previous knowledge regarding stroke						
Yes	55	39	128	137.31	2	0.00*
No	67	23	12			

## Discussion

The current study indicates that 26.85% of hypertensive patients had poor awareness, 41.66% had fair awareness (mean score 13.84±5.92), and 31.48% demonstrated good awareness regarding stroke. These findings are comparable to a study by Misgana et al. [11], which reported that approximately 30% of participants had low awareness of stroke symptoms, while around 40% exhibited moderate awareness. Both studies highlight the prevalence of limited knowledge about stroke, emphasizing the need for targeted educational initiatives to improve understanding, particularly in recognizing risk factors and early warning signs to facilitate timely intervention.

The study reveals varying levels of awareness across different stroke-related domains, with the highest awareness in treatment and prevention (75.7%) and warning signs and symptoms (73.7%), while awareness of risk factors (31.25%) and diagnostic tests (50.5%) was relatively low. These findings align with a study by Pathak et al. [12], which reported that 70% of participants recognized stroke symptoms, but only 35% were aware of its risk factors. Both studies highlight a consistent gap in knowledge regarding stroke risk factors and diagnostic approaches, underscoring the need for comprehensive educational programs to improve awareness across all domains, particularly in understanding stroke causes and early detection methods.

The present study highlights that approximately one-third of hypertensive patients were aware of one to two risk factors for stroke, while three-fourths recognized key signs and symptoms, such as imbalance of the body, one-sided body weakness, difficulty in speech, and vision-related problems. These findings are consistent with a study by Webb and Werring [13], which reported that 35% of participants had knowledge of stroke risk factors and 70% were aware of its symptoms. Similarly, Silva et al. [14] found that 68% of participants identified at least one symptom, such as headache or hemiparesis, and 85.4% recognized at least one risk factor, including hypertension or smoking.

The present study explores personal and clinical characteristics associated with the level of stroke awareness among hypertensive patients. Significant factors influencing awareness included age, education, occupation, social background, duration of hypertension, BMI, and prior knowledge of stroke (p<0.05). For instance, participants aged 41-60 years and those with formal education showed higher awareness, while those from urban areas exhibited better awareness than their rural counterparts. These findings align with a study by Vujosevic et al. [15], which identified education level, urban residency, and previous exposure to stroke information as key determinants of awareness. Similar to this study, Pandian et al. also reported a significant association between BMI and awareness levels [8].

This study, conducted within a single institution, has limited generalizability. The findings indicate that hypertensive patients have inadequate awareness of stroke causes and risk factors. The results suggest that disseminating information through various channels such as media, magazines, newspapers, role plays, pamphlets, informational booklets, and health education can significantly enhance patients' understanding of stroke and its prevention.

## Conclusions

The findings of this study highlight a significant gap in stroke awareness among hypertensive patients, particularly regarding risk factors and diagnostic methods, despite relatively higher recognition of symptoms and prevention strategies. With 26.85% of patients exhibiting poor awareness and only 31.48% demonstrating good knowledge, these results underscore the urgent need for targeted educational interventions. The study's alignment with previous research further reinforces the widespread nature of this knowledge deficit. Key determinants such as age, education, occupation, social background, BMI, and prior stroke awareness were found to influence knowledge levels, emphasizing the role of personalized health education strategies. Patients with formal education and urban residency demonstrated higher awareness, highlighting the need for outreach programs tailored to rural populations and those with lower education levels.

Addressing these gaps through regular awareness campaigns and structured educational programs can enhance early recognition of stroke symptoms, improve preventive behaviors, and ultimately reduce stroke-related morbidity and mortality. Future initiatives should focus on empowering hypertensive patients with comprehensive knowledge, fostering proactive health-seeking behaviors, and bridging the existing disparities in stroke awareness.
